# The Src family kinase inhibitor dasatinib delays pain-related behaviour and conserves bone in a rat model of cancer-induced bone pain

**DOI:** 10.1038/s41598-017-05029-1

**Published:** 2017-07-06

**Authors:** Camilla Kristine Appel, Simone Gallego-Pedersen, Line Andersen, Sophie Blancheflor Kristensen, Ming Ding, Sarah Falk, Manasi Sayilekshmy, Charlotte Gabel-Jensen, Anne-Marie Heegaard

**Affiliations:** 1 University of Copenhagen, Faculty of Health and Medical Sciences, Department of Drug Design and Pharmacology, Copenhagen, 2100 Denmark; 2 Odense University Hospital, University of Southern Denmark, Institute of Clinical Research, Department of Orthopaedics and Traumatology, Odense, 5320 Denmark; 3 University of Copenhagen, Faculty of Health and Medical Sciences, Department of Pharmacy, Copenhagen, 2100 Denmark

**Keywords:** Bone cancer, Preclinical research, Chronic pain

## Abstract

Pain is a severe and debilitating complication of metastatic bone cancer. Current analgesics do not provide sufficient pain relief for all patients, creating a great need for new treatment options. The Src kinase, a non-receptor protein tyrosine kinase, is implicated in processes involved in cancer-induced bone pain, including cancer growth, osteoclastic bone degradation and nociceptive signalling. Here we investigate the role of dasatinib, an oral Src kinase family and Bcr-Abl tyrosine kinase inhibitor, in an animal model of cancer-induced bone pain. Daily administration of dasatinib (15 mg/kg, p.o.) from day 7 after inoculation of MRMT-1 mammary carcinoma cells significantly attenuated movement-evoked and non-evoked pain behaviour in cancer-bearing rats. Radiographic - and microcomputed tomographic analyses showed significantly higher relative bone density and considerably preserved bone micro-architecture in the dasatinib treated groups, suggesting a bone-preserving effect. This was supported by a significant reduction of serum TRACP 5b levels in cancer-bearing rats treated with 15 mg/kg dasatinib. Furthermore, immunoblotting of lumbar spinal segments showed an increased activation of Src but not the NMDA receptor subunit 2B. These findings support a role of dasatinib as a disease modifying drug in pain pathologies characterized by increased osteoclast activity, such as bone metastases.

## Introduction

Pain is a common, feared and serious complication of cancer occurring in more than 70% of patients with advanced stage cancer^1–3^. Bone cancer most often arises when cancerous cells from a primary tumour e.g. breast, prostate or lung metastasize to the bone^4^. In the clinic, patients with cancer-induced bone pain describe the pain as heterogeneous and gradually increasing with disease progression^5^. Current treatment options include non-steroidal inflammatory drugs (NSAIDS), opioids and antiresorptive agents such as bisphosphonates^1, 6^. Although opioids have a key role in pain management, opioids are associated with the development of tolerance, thereby increasing the required dose, leading to severe adverse effects, including nausea, dizziness, constipation, respiratory depression, sedation as well as the risk of addiction^6^. Other treatment options include radiotherapy or radioisotopes^7^. Radiotherapy has proven to be one of the most efficient treatments for relieving pain in patients suffering from uncomplicated bone metastases. Up to 60% of patients receiving treatment report significant pain relief and 25% experience complete pain relief^7^. However, there is a need for improved treatment options and the mechanisms underlying cancer-induced bone pain is still not fully understood.

Cancer-induced bone pain contains components of both neuropathic and inflammatory pain but also has its own distinctive characteristics making it a unique pain state^8, 9^. When the tumour grows in the bone it damages the surrounding nerves and tissue; produces a wide range of cytokines and growth factors; stimulates inflammatory infiltration, and increases bone degradation^10, 11^. Simultaneously, a reorganization of the central nervous system in the areas receiving information from the cancer-bearing area takes place, driving a central sensitization, i.e. increased response and activity of dorsal horn neurons to nociceptive inputs^12, 13^. The unique neurochemical identity of cancer-induced bone pain may explain why cancer pain often is refractory to traditional analgesics^14–16^. One potential future analgesic target is the non-receptor tyrosine kinase Src which is a member of the Src kinase family. Src is a widely expressed kinase potentially involved in several of the processes leading to cancer-induced bone pain^17–20^. Src is encoded by the *Src* gene and was the first identified proto oncogene. Src has been implicated in cancer growth, angiogenesis and metastasis^17^ and increased expression and activity of Src has been correlated to advanced cancer and poor prognosis in a variety of human cancers^21, 22^. Furthermore, Src is a key regulator of normal bone homeostasis^18, 23^. It is crucial for the resorbing function of osteoclasts^23^ and targeted deletion of the Src gene in mice yields osteopetrotic mice, i.e. mice with increased bone mass^18^. Finally, Src family kinases are ubiquitously expressed in the central nervous system and abundant in neurons^24^. Src is involved in several fundamental signalling pathways, e.g., epidermal growth factor (EGF), extracellular regulated kinase (ERK) and EphrinB2^25, 26^. In regards to pain pathologies, Src has been demonstrated to play an important role, due to its attachment to the N-methyl-D-aspartate (NMDA) receptor 2B subunit (NR2B) in the NMDA receptor complex. Phosphorylation of Src induce phosphorylation of the NMDA receptors which up-regulates the receptor activity and increases its channel open time and its opening probability^27–29^. It has been shown that activation of Src contributes to inflammatory pain through phosphorylation of the NR2B in rats^30, 31^. Furthermore, Liu and colleagues reported in 2008 that blocking the interaction between Src and the NMDA receptor complex with a peptide mimicking the amino acid sequence of the unique domain of Src ameliorates inflammatory and neuropathic pain^32, 33^ and recently Felice *et al*. reported that the multi-kinase inhibitor saracatinib inhibits thermal hyperalgesia in cancer-induced bone pain^34^. Thus employing a Src kinase inhibitor could be an optimal way of targeting cancer-induced bone pain in a multi-targeted manner. Dasatinib is a multi-kinase inhibitor approved for the treatment of chronic myeloid leukaemia, imatinib-resistant or -intolerant chronic myelogenous leukaemia and Philadelphia positive acute lymphoblastic leukaemia^35^. Dasatinib is a highly potent inhibitor of the Src kinase family as well as other kinases such as BCR-Abl, Fyn, c-Kit, platelet-derived growth factor receptor (PDGFR) alpha and –beta, and the ephrin receptor kinase^36^. Pre-clinically dasatinib has been shown to inhibit tumour growth^37–39^, osteoclast function^40^ and induce osteoblast activity^41, 42^. Clinically the effect of dasatinib has been studied in solid tumours^43, 44^ and in combination with zoledronic acid for the treatment of breast-cancer bone metastasis^45^. The latter clinical study concluded that combination therapy was beneficial in hormone receptor positive breast cancers in regards to bone response and tumour growth^45^. Dasatinib is already clinically approved for treatment of chronic myeloid leukaemia and is a well-tolerated therapy. We hypothesize that dasatinib treatment could attenuate cancer-induced bone pain. Therefore, using a rat model of cancer-induced bone pain, we sought to determine whether inhibition of Src family tyrosine kinases by dasatinib would decrease tumour burden, decrease bone degradation, and reduce pain-related behaviours. Using a combination of behavioural, imaging, and immunoblotting assays we show that dasatinib acts as a disease modifier that delays onset of cancer-induced bone pain by preserving bone mass.

## Results

### High dose dasatinib delays onset of pain-related behaviours

**Figure 1:**
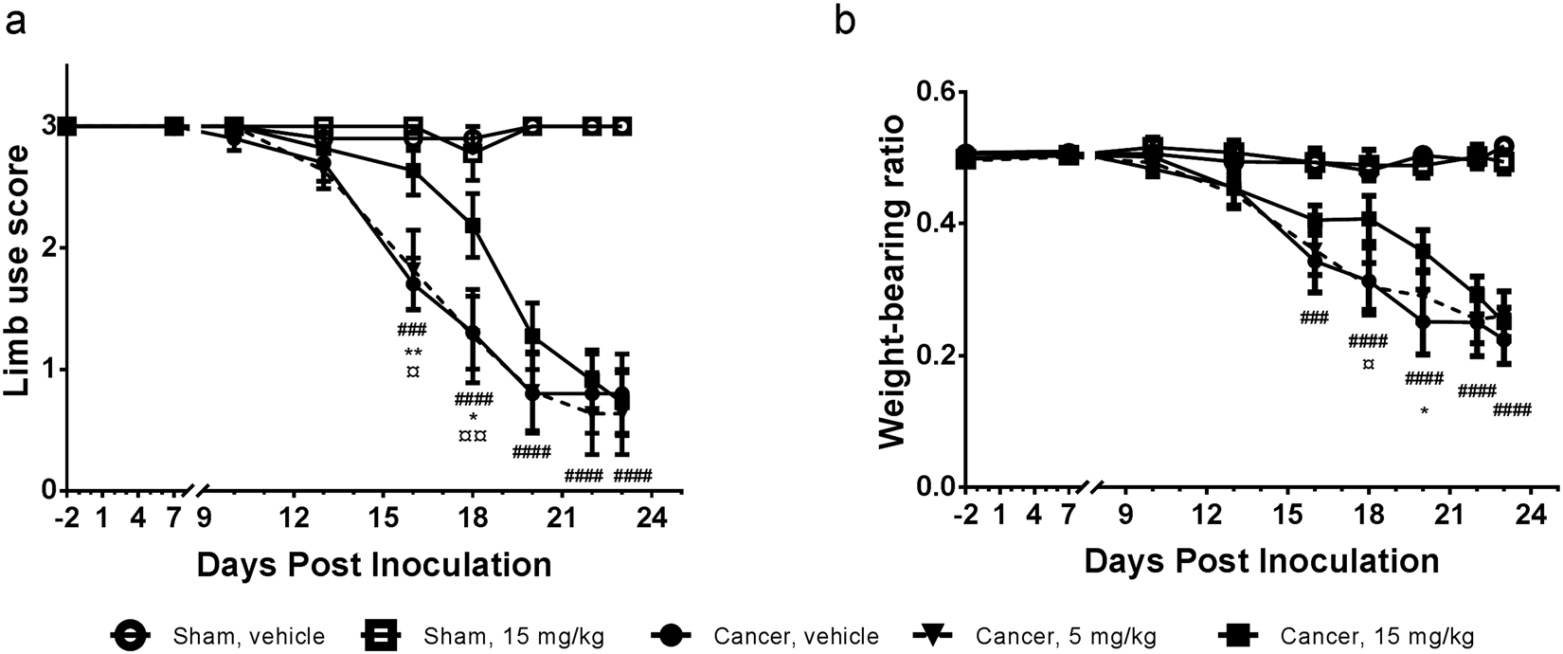
Chronic oral treatment with high dose dasatinib delays onset of skeletal pain-related behaviour in the rat model of cancer-induced bone pain. (**a**) High dose (15 mg/kg) dasatinib induces an anti-nociceptive effect on post inoculation day 16 and 18 compared to the control vehicle group, demonstrating a delay in onset of movement evoked pain behaviour. (**b**) Weight-bearing deficits are significantly delayed in the high dose dasatinib group compared to the vehicle treated control. Two-way repeated-measurement ANOVA followed by Bonferroni’s multiple comparison test revealed statistically significant differences among the groups with regard to pain-related behaviors. Data are displayed as mean ± SEM, n = 10–11. ^#^Cancer-operated group treated with vehicle compared to sham-operated group treated with vehicle; *Cancer-operated rats treated with 15 mg/kg dasatinib compared to the vehicle treated cancer-operated group; ^¤^Cancer-operated rats treated with 15 mg/kg dasatinib compared to cancer-operated rats treated with 5 mg/kg dasatinib.

To test the analgesic potential of dasatinib on cancer-induced bone pain, the limb use test and the weight-bearing test was used to assess pain-related behaviours. Animals inoculated with mammary cancer cells and treated with vehicle developed a significant reduction in limb use score from day 16 (p = 0.0001). Daily oral administration of high dose (15 mg/kg) dasatinib significantly delayed the onset of movement-evoked pain demonstrated by a significant difference in limb use score on day 16 (p = 0.005) and 18 (p = 0.0103) compared to the vehicle treated cancer-bearing group (Fig. 1a). In addition, on day 16 (p = 0.018) and 18 (p = 0.0053) there was a significant difference between the high and low dose dasatinib cancer-bearing groups in the limb use test. These data were supported by the weight-bearing test. Cancer-bearing animals treated with vehicle demonstrated a significant weight-bearing deficit from day 16 post inoculation compared to sham-operated rats (p = 0.0005). Rats treated with high dose dasatinib had a significantly improved weight-bearing ratio compared to both low dose treated rats (p = 0.0351, day 18) and vehicle treated rats (p = 0.0296, day 20) (Fig. 1b). Low dose (5 mg/kg) dasatinib had no effect on development of pain-related behaviour. The two sham groups did not show pain-related behaviour in any of the two tests.

### Dasatinib affects spinal Src phosphorylation but not NMDA receptor subunit 2B phosphorylation

**Figure 2:**
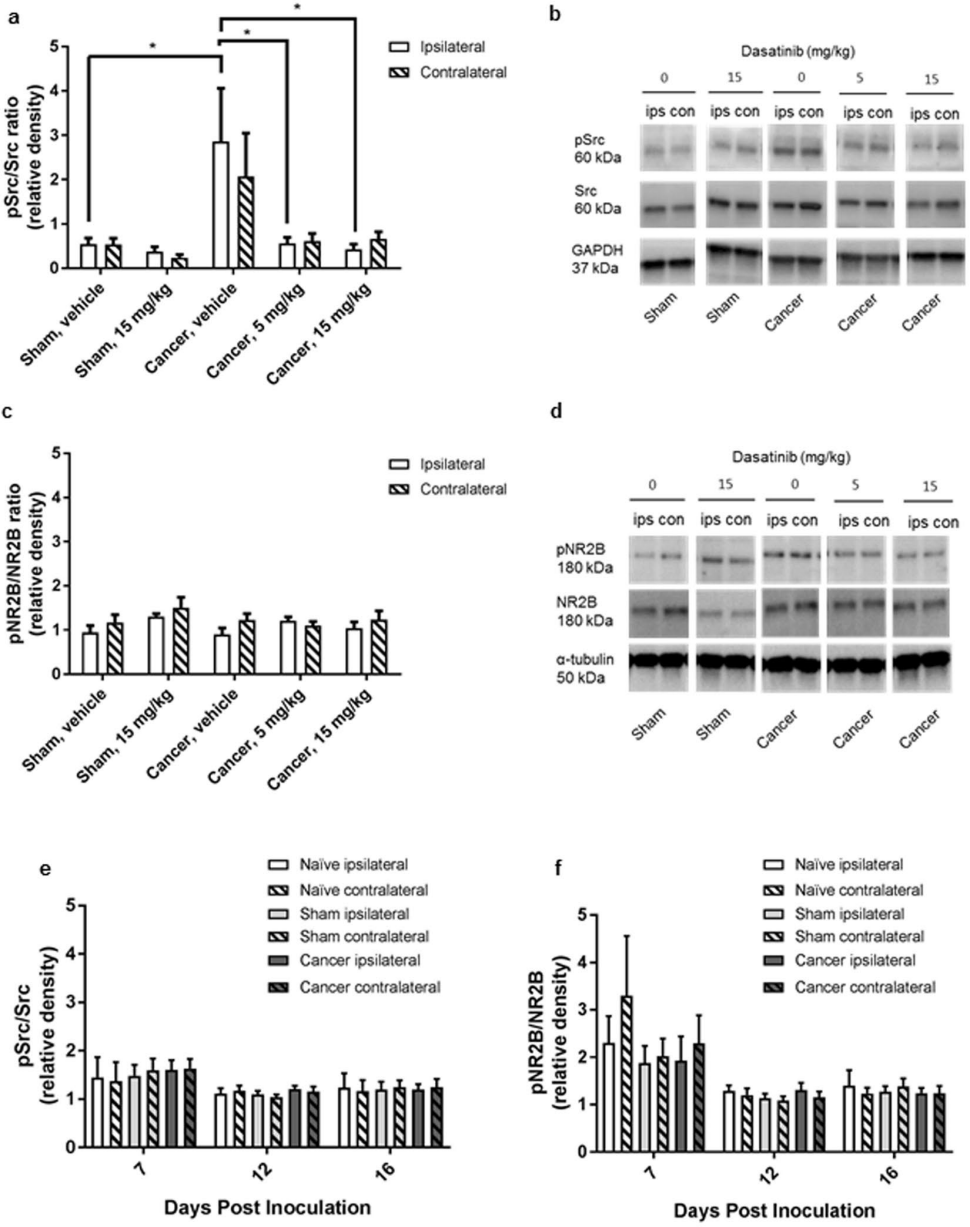
Increased spinal cord Src phosphorylation in cancer-induced bone pain is blocked by dasatinib. Ratios of spinal expression of phosphorylated Src (pSrc) to total Src (**a**,**e**) and phosphorylated NMDA receptor subunit 2B (pNR2B) to total NR2B (NR2B) (**b**,**f**). (**a**) Cancer-induced bone pain induces an increase of spinal pSrc/Src ratio expression at the time of euthanasia. Both high and low dose dasatinib significantly decreases the level of phosphorylation of Src compared to vehicle treated control. B: Representatives of Western Blot. (**c**) No significant changes of spinal pNR2B/NR2b ratio expression were observed between the five treatment groups at the time of euthanasia. (**d**) Representatives of Western Blot. (**e**,**f**): Timeline study of cancer-induced bone. No significant change in the expression of spinal pSrc/Src (**e**) or pNR2B/NR2B (**f**) is observed during development of cancer-induced bone pain in the female rat. One-way ANOVA followed by Bonferroni’s multiple comparison test. Data are displayed as mean ± SEM, n = 4–10. The blots displayed are cropped and the full-length blots are available in the Supplementary Information file.

To investigate if dasatinib inhibits phosphorylation of Src in the spinal cord western blots were performed. Cancer-bearing animals treated with vehicle had a significant increase in Src phosphorylation compared to vehicle treated controls (p = 0.0374). Dasatinib significantly reduced the pSrc/Src ratio back to baseline levels in both the high (p = 0.0204) and low (p = 0.0256) treatment group (Fig. 2a,b); however this did not translate into an effect on phosphorylation of the NR2B. No difference was detected in phosphorylation of NR2B in the dasatinib treated groups compared to the control groups in the advanced disease state (Fig. 2c,d). To further test whether cancer-induced bone pain is associated with an increased phosphorylation of Src and the NR2B during early disease development, western blot analyses were performed on spinal cords from untreated cancer-bearing and sham animals on day 7, 12 and 16 post inoculation. The Src level was unchanged (P > 0.05) between cancer and sham groups (Fig. 2b), making the pSrc/Src level indicative of the amount of Src phosphorylation. No difference in pNR2B/NR2B or pSrc/Src ratio was detected between the two groups as disease progressed (Fig. 2e,f) or at advanced-stage disease (Fig. 2a and c).

### Dasatinib significantly preserves bone mass

**Figure 3:**
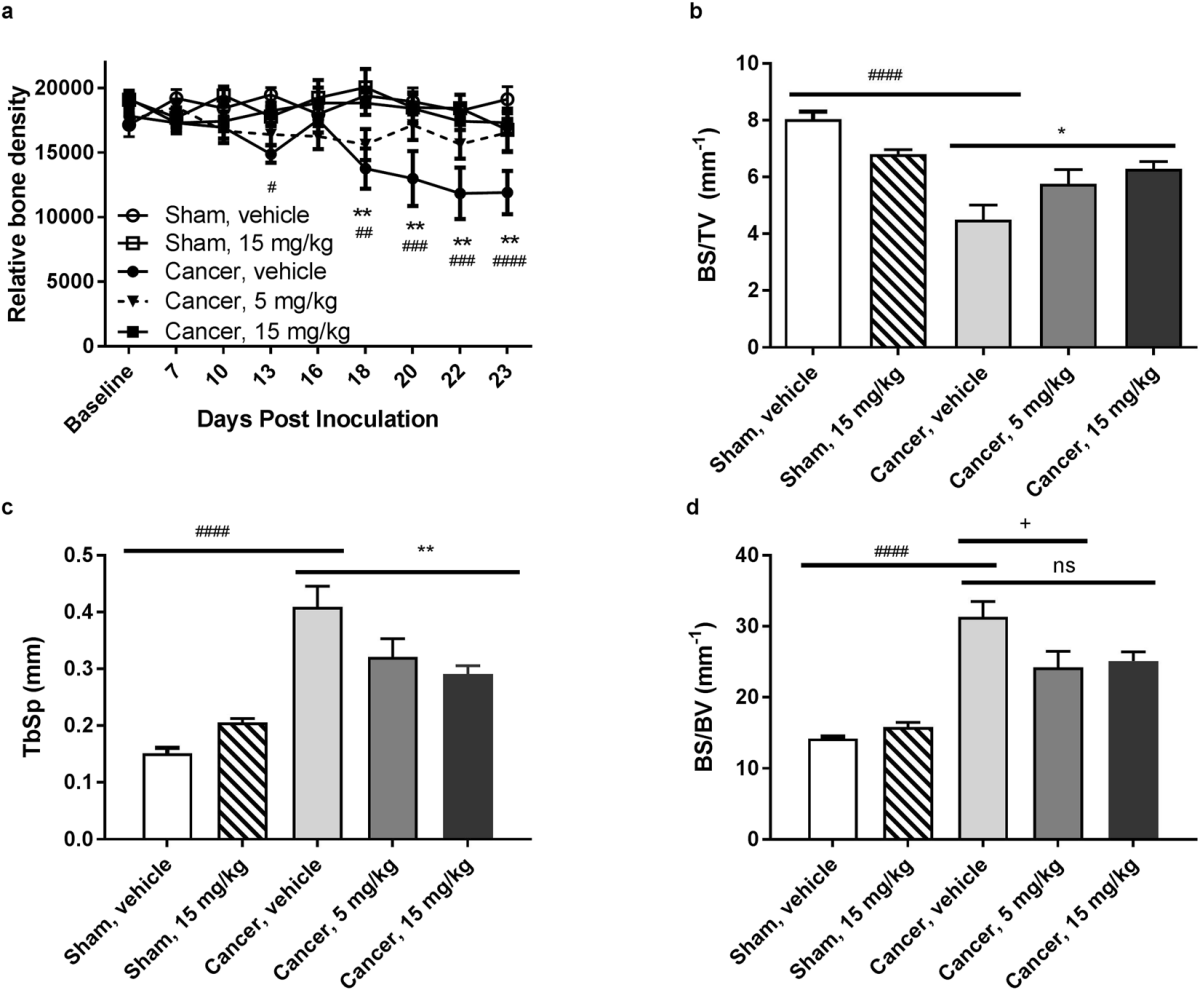
Dasatinib conserves bone mass in cancer-bearing rats. (**a**) Two-way repeated-measurement ANOVA followed by Bonferroni’s multiple comparison test showed that the cancer vehicle group is significant decrease in relative bone density compared to the sham operated group on day 13, 18−23. High dose (15 mg/kg) dasatinib preserved bone density throughout the experiment compared to vehicle treated cancer control. (**b**–**d**) Microcomputed tomographic analysis of cancer-bearing tibia analysed by a one-way repeated-measurement ANOVA followed by Bonferroni’s multiple comparison test. (**b**) High dose dasatinib preserves the bone surface density compared to the cancer vehicle group. (**c**) High dose dasatinib treatment lowers the trabecular separation significantly compared to the cancer vehicle control group. (**d**) Low dose dasatinib decreases the bone surface-to-volume ratio compared to cancer vehicle control. Data are displayed as mean ± SEM, n = 10–11. ^#^Cancer-bearing group treated with vehicle compared to sham group treated with vehicle; ^*^Cancer-bearing rats treated with 15 mg/kg dasatinib compared to the vehicle treated cancer-bearing group; ^+^Cancer-operated rats treated with vehicle compared to cancer-operated rats treated with 5 mg/kg dasatinib.

**Figure 4:**
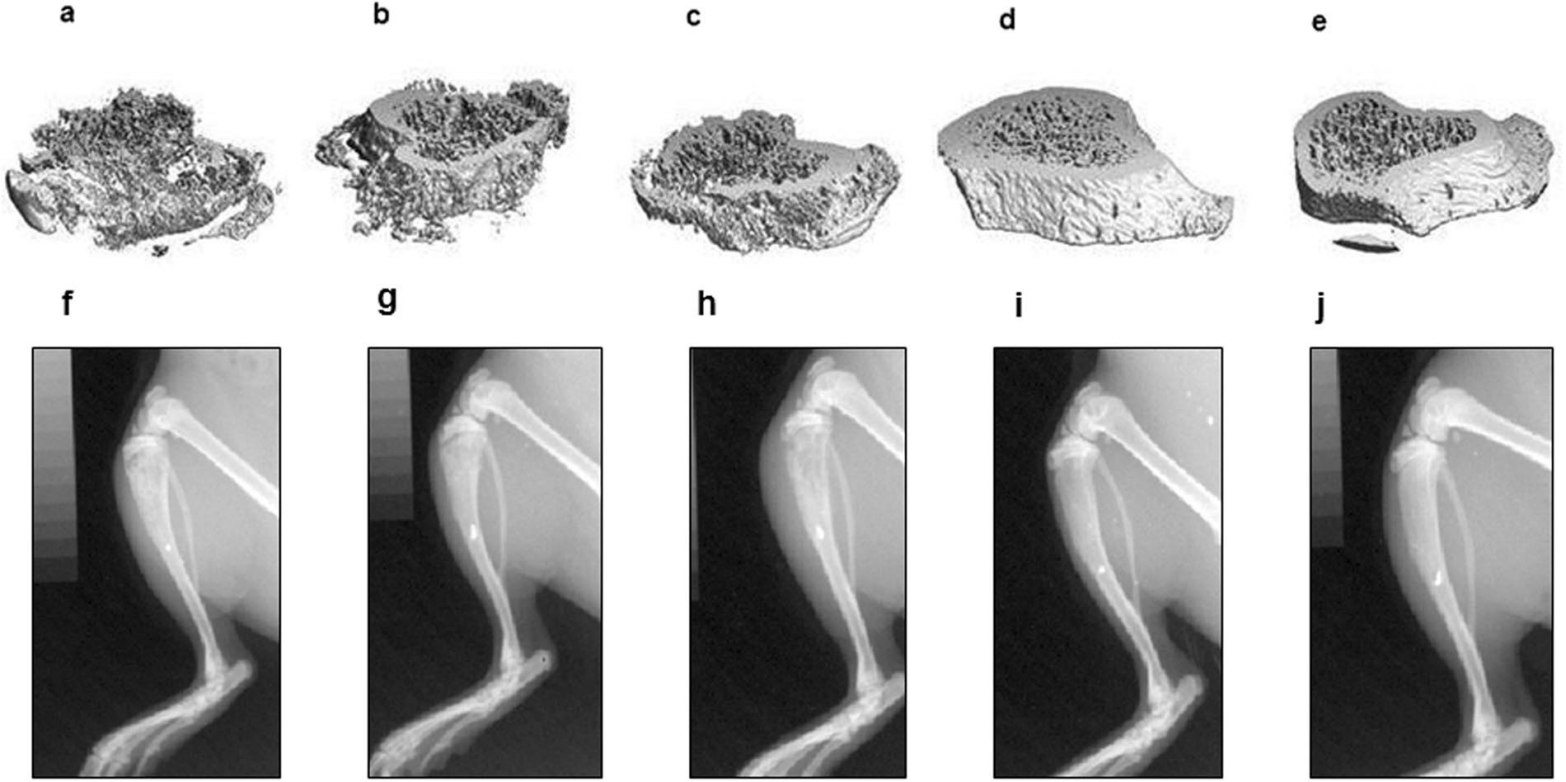
Micro-CT and x-ray images illustrating the proximal part of the tibia of the five different treatment groups. Micro CT image captured post-mortem of (**a**) cancer-operated and treated with vehicle; (**b**) Cancer-operated and treated with 5 mg/kg dasatinib; (**c**) Cancer-operated and treated with 15 mg/kg dasatinib; (**d**) Sham-operated and treated with vehicle; and (**e**) Sham-operated and treated with 15 mg/kg dasatinib. X-ray images captured on day 22 post inoculation f) cancer-operated and treated with vehicle; (**g**) Cancer-operated and treated with 5 mg/kg dasatinib; (**h**) Cancer-operated and treated with 15 mg/kg dasatinib; (**i**) Sham-operated and treated with vehicle; and (**j**) Sham-operated and treated with 15 mg/kg dasatinib.

To test if the observed delay in pain development was mediated by a bone preserving effect of dasatinib, x-ray photos of the ipsilateral tibia were captured on the day of baseline measurements and on day 7, 10, 13, 16, 18, 20, 22 and 23. High dose dasatinib significantly preserved the relative bone density on day 18–23 (p < 0.01, Fig. 3a) compared to the cancer vehicle group, suggesting that dasatinib prevents bone degradation induced by the cancer. No significant difference in bone density was observed between the two cancer-bearing groups treated with different doses of dasatinib or between the two sham-operated groups (p > 0.05). The increase in relative bone density for the cancer-operated group treated with high dose dasatinib was supported by µCT analyses of the bone (Fig. 4a–e). Post mortem micro-architectural analysis of the proximal part of the ipsilateral tibia showed a significantly higher bone surface density of the high dose dasatinib treated cancer-bearing animals compared to vehicle treated cancer-operated animals (p = 0.013, Fig. 3b) as well as significantly lower trabecular separation (p = 0.0077, Fig. 3c). In addition, cancer-operated rats treated with 5 mg/kg dasatinib had significantly lower bone surface-to-volume ratio compared to control (p = 0.0242, Fig. 3d). There was no significant difference between the high and low dasatinib dose groups in any of the micro-architectural parameters investigated (p > 0.05, Fig. 3b–d).

### High dose dasatinib significantly decreased serum TRACP 5b levels

**Figure 5:**
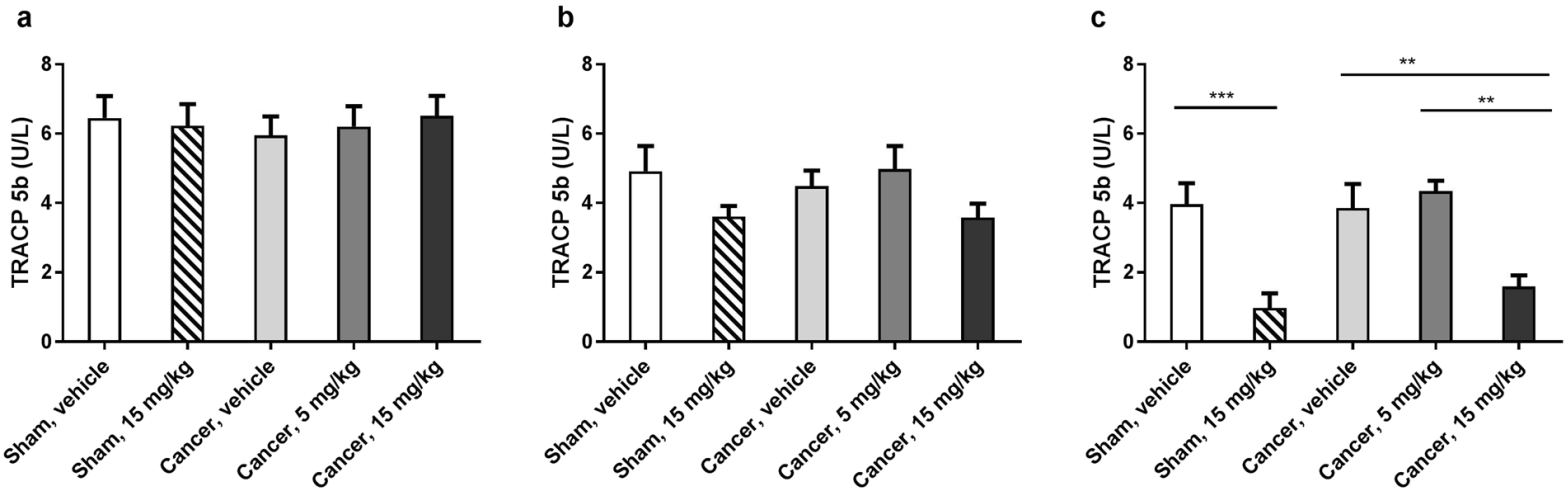
High dose dasatinib significantly lowers serum levels of TRACP 5b compared to vehicle treated controls. (**a**) There is no significant difference between the groups on day 6, i.e. one day before starting dasatinib treatment. (**b**) There is no significant difference between the groups on day 12, i.e. after 6 days of dasatinib treatment. (**c**) Serum TRACP 5b is significantly decreased in the high dose dasatinib treated groups compared to vehicle treated controls on day 23 post inoculation. Low dose dasatinib does not influence the serum TRACP 5b values compared to vehicle treated control. Ordinary one-way repeated-measurement ANOVA followed by Bonferroni’s multiple comparison test. The results are shown as the mean ± SEM, n = 10–11.

Serum tartrate-resistant acid phosphatase 5b was measured on days 6, 12 and 23 post inoculation, hence before and following 6 and 12 days of dasatinib treatment, to evaluate the effect of dasatinib on the number of bone-resorbing osteoclasts. High dose dasatinib treatment of cancer-bearing animals significantly decreased serum TRACP 5b levels on day 23 compared to the vehicle treated controls (p = 0.017, Fig. 5c). Also on day 23 the dasatinib treated sham group had a significant decrease in serum TRACP 5b levels compared to the vehicle treated sham group (p = 0.0008). Low dose dasatinib had no significant effect on TRACP 5b levels when compared to the vehicle treated cancer controls on day 23 (p > 0.999, Fig. 5), and was significantly different from the high dose treated cancer group (p = 0.0014). The cancer-operated vehicle group did not show increased TRACP 5b levels compared to the sham-operated vehicle group on any of the test days. There was no significant difference between the groups on day 6, i.e. the day before initiation of dasatinib treatment (p > 0.999, Fig. 5a). On day 12, after 6 days of dasatinib treatment, however a tendency for the high dose dasatinib to reduce serum levels of TRACP 5b compared to vehicle treatment (p = 0.97, Fig. 5b) was observed.

### Dasatinib inhibits proliferation of the MRMT1-*luc2* mammary carcinoma cells *in vitro*

**Figure 6:**
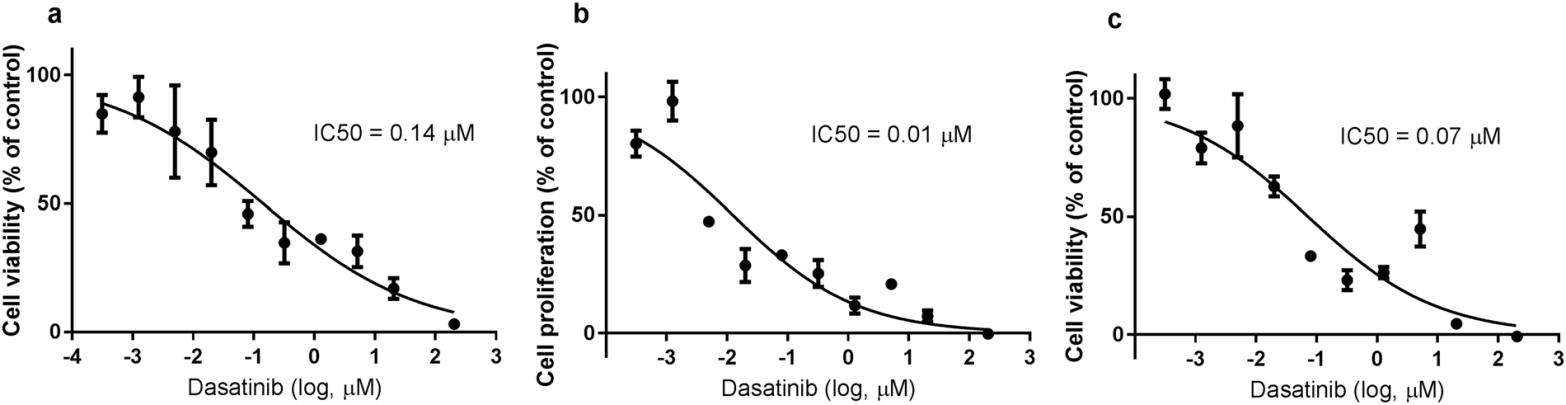
Dasatinib inhibits cell proliferation in MRMT1-*luc2* cells in a dose-dependent manner *in vitro*. MRMT1-*luc2* cells were treated with increasing concentrations of dasitanib for 48 h and assayed by the (**a**) MTT assay, (**b**) the BrdU assay, and (**c**) the RealTime-Glo™ assay. IC50, the concentration needed to inhibit viability or proliferation by 50. Data were normalized to cells treated with 0.1% DMSO (negative controls, 100% activity) and blank controls, e.g. wells without cells (0% activity) and expressed as percentage of these controls. Data are displayed as mean ± SEM. Normalized response is compared to common log of dasatinib concentration (µM) and the IC50 calculated using a variable slope. Data from the MTT assay represents one of three independent experiments done in quadruplicates. Data from the BrDU and RealTime-Glo™ assays represents one experiment done in triplicates to confirm the results from the MTT assay.

The effect of dasatinib on tumour proliferation was tested *in vitro*. Cells were treated with increasing doses (3·10^−4^–205 µM) of dasatinib for 48 hours and the metabolic activity of the cells investigated. There was a significant decrease in viable cells compared to the untreated control as the concentration of dasatinib increased (Fig. 6a–c). The IC_50_ value for the MRMT1-*luc2* cell line in the MTT assay was 0.142 µM with a 95% confidence interval (95CI) of [0.061; 0.334]. The results were supported by the BrdU assay and RealTime-Glo™ MT Cell Viability Assay, with IC50 values of 0.012 µM 95CI [0.006; 0.026] and 0.072 µM 95CI [0.030; 0.173].

### Dasatinib treatment does not affect MRMT1-*luc2* mammary carcinoma cell bioluminescent signal *in vivo* compared to vehicle treated rats

**Figure 7:**
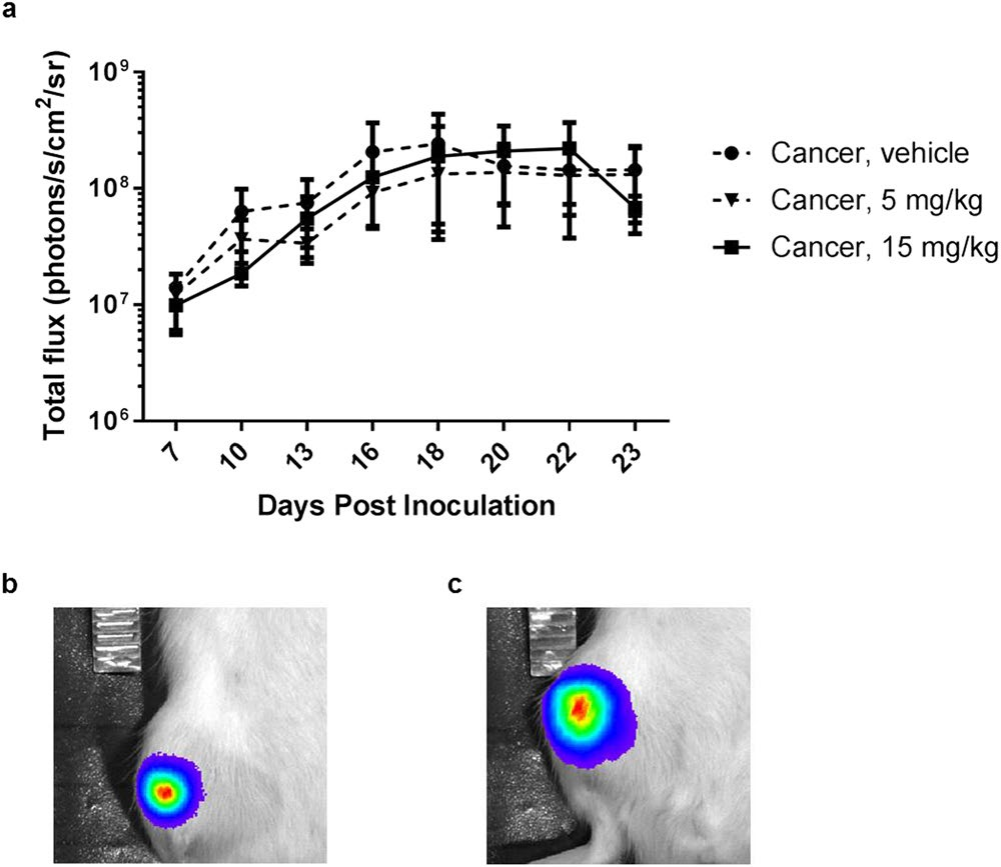
Bioluminescence signal from cancer cells *in vivo*. (**a**) The signal increases in all groups during the first 16 days until it reaches a plateau. Two-way repeated-measurement ANOVA followed by Bonferroni’s multiple comparison test did not reveal any statistically significant differences between the treatment groups when compared to vehicle on any of the test days (p < 0.05). Data are displayed as mean ± SEM, n = 10–11. Bioluminescent overlay on bright field images (**b**) example of low bioluminescent signal 7 days post inoculation and (**c**) example of high bioluminescent signal 19 days post inoculation.

To test if dasatinib affects the bioluminescent tumour signal *in vivo* bioluminescence was measured over time starting from day 7 post cancer cell inoculation. The bioluminescent signal increased over time until it reached a plateau around day 16 (Fig. 7a–c). No significant difference in bioluminescent signal was observed among the three cancer-operated groups on any of the test days (p > 0.999, Fig. 7a).

### Chronic treatment of 30 mg/kg dasatinib induces severe adverse events

**Figure 8:**
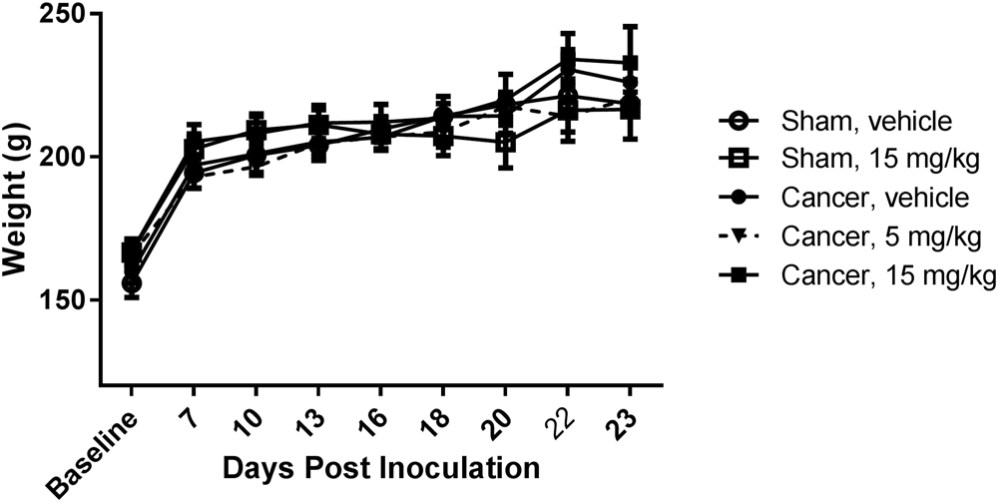
Dasatinib did not lead to loss of body weight. Two-way repeated-measurement ANOVA followed by Bonferroni’s multiple comparison test did not reveal any statistically significant differences among cancer bearing and sham-operated animals or between the dasatinib-treated groups (p < 0.05). Data are displayed as mean ± SEM, n = 10–11.

The general health status of the rats was assessed by determining the use of the cancer-bearing limb, grooming behaviour and by measuring body weight every third day (Fig. 8). In the sham-operated group receiving high dose dasatinib (15 mg/kg), 4 out of 10 animals exhibited adverse events (e.g. gastro intestinal side effects and fatigue) resulting in euthanasia of one rat on day 16 and three on day 20. A higher dose of 30 mg/kg, previously tested by Tokuhisa *et al*., 2014 for advanced extremity melanoma^46^, was initially included in the study. However, the dose lead to severe adverse events (e.g. distension of the gastrointestinal tract with gas/fluid/digesta, weight loss and fatigue) in both sham and cancer-operated animals and the animals were euthanized on day 15 or 16. Data from the 30 mg/kg rats have hence not been included in the data analyses. The dose of 5 mg/kg dasatinib did not lead to any adverse events.

## Discussion

Despite improvement in treatment options, many patients with advanced- or metastatic cancer experience unsatisfactory pain relief which severely compromises their quality of life^2, 14^. Various pre-clinical studies have demonstrated that dasatinib significantly inhibits tumour growth and osteoclast function, and increase osteoblast activity. Thus, our original hypothesis was that inhibition of Src family tyrosine kinases by dasatinib would reduce pain-related behaviour through a decrease in NMDAR mediated central sensitization, tumour burden and bone degradation. Indeed, the current study supports a potential therapeutic role for dasatinib in cancer-induced bone pain. Administration of 15 mg/kg dasatinib efficiently delayed onset of movement-evoked and non-evoked pain compared to the vehicle group in the MRMT-1 model of cancer induced bone pain.

To elucidate the mechanism underlying the analgesic effect of dasatinib kinase inhibition, we tested the hypothesis that the delay in pain-related behaviour was induced by inhibition of Src mediated phosphorylation of NR2B in the spinal cord. In the dorsal horn, Src holds a critical regulatory role in the generation and maintenance of central sensitization through activation of NMDAR function by phosphorylation of the NR2B subunit^31, 47^. The NR2 subunits of the NMDA receptor complex are involved in binding of glutamate and determine the receptor kinetics. The NR2B subunit is predominantly expressed in the superficial dorsal horn and has been identified as the key Src-mediated-tyrosine-phosphorylated subunit in the NMDA receptor complex^33^. It has been proposed to be involved in development of spinal plasticity and hyperalgesia after inflammatory and neuropathic pain^30, 48^ as well as cancer-induced bone pain^49–51^. Liu *et al*. reported that blocking the Src-dependent phosphorylation of NMDA through an anchoring protein reversed inflammatory and neuropathic pain^32^. In this study both doses of dasatinib are able to block Src phosphorylation in the spinal cord, however, this effect does not translate into a significant effect on the level of NMDA receptor 2B phosphorylation at Tyr1472. Similarly, results by Felice *et al*.^34^ show increased pSrc/Src in the MRMT-1 male rat model of cancer-induced bone pain but did not demonstrate an effect on NR2B phosphorylation. Overall, our data indicates that the analgesic effect of high dose dasatinib treatment does not result from inhibiting Src phosphorylation at the spinal cord level, but rather from a disease modifying effect on the bone and possibly an anti-proliferative effect on the tumour, causing an indirect anti-nociceptive effect. However, even though dasatinib has high selectivity for Src and the other SFKs, e.g. Fyn, Lck, Yes as well as the Bcr-Abl kinase, dasatinib is a multi-kinase inhibitor and also targets several other kinases such as c-kit, PDGFRβ, p38 mitogen-activated protein kinase, Her1, Her2 and FGFR-1^36^. One or several of these kinases may convey specific or unspecific anti-nociception. c-kit is for example expressed in lamina I and II of the dorsal horn and reported to be involved in development of persistent nociceptive pathways upstream from Src^52, 53^. Likewise, p38 mitogen-activated protein kinase and FGFR-1 are also reported to have roles in different pain pathologies, including cancer-induced bone pain^54–56^. It is therefore not possible to exclude a non-Src mediated anti-nociceptive effect of dasatinib.

Dasatinib has been demonstrated to have an inhibitory effect on the growth of several cancer types both *in vitro* and *in vivo* ^39, 57–59^. Korenckji and others reported that dasatinib inhibits prostate cancer growth in bone in mice^60^. We therefore hypothesized that the delay in onset of pain was mediated by a lower tumour burden in the dasatinib-treated animals. The effect of dasatinib on the MRMT1-*luc2* cell line has not previously been evaluated. *In vitro* treatment of MRMT-1 mammary carcinoma cells with dasatinib significantly inhibited cell proliferation and viability in the MTT assay in a concentration-dependent way. As the reliability of the MTT assay for testing similar drugs, such as imatinib, has been questioned^61, 62^, the *in vitro* effect of dasitinib was in addition tested using the RealTime-Glo™ MT Cell Viability Assay and BrdU assay. Both assays supported an inhibitory effect of dasatinib on MRMT-1 cancer cell viability and proliferation *in vitro*. However, *in vivo* bioluminescent imaging did not suggest any decrease in signal after treatment with dasatinib. In all groups the bioluminescent signal increased in the initial 16 days before reaching a plateau. When the signal reach the plateau phase it is likely that the value of bioluminescence is limited when estimating tumour volume of large, necrotic tumours due to lack of oxygen and ATP, and hence it cannot be excluded that dasatinib had an anti-proliferative effect on the tumour burden *in vivo*. Overall, the reported results on the MRMT1-*luc2* cell line *in vitro* and *in vivo* suggests a correlation with previous data where dasatinib was shown to inhibit proliferation of cancer cell lines *in vitro* ^39, 63^, however an effect that did not translate *in vivo* ^63^ *or* to the clinic^45, 64^.

Lastly, we tested if the delay in pain-related behaviours was mediated by a bone mass preserving effect of dasatinib. In accordance with this hypothesis we here report a bone preserving effect of dasatinib supported by both x-ray images during the disease progression and µCT analyses post mortem. Moreover, high dose dasatinib induced a decrease in the osteoclast marker TRACP 5b which reflects the number of osteoclasts^65^. This is supported by clinical data^44^ reporting a decrease in bone resorption markers, NTx and TRACP-5b of 46.3% and 22.2%, respectively, after treatment with dasatinib and zoledronic acid. Previous results in *Src* knockout mice demonstrated that after depletion of *Src* the function of osteoclasts were diminished because full maturation of the osteoclast was inhibited. However, depletion of *Src* does not decrease the amount of osteoclasts in the bone microenvironment^18, 23^. This indicates that the suggested reduction in osteoclast number seen after treatment with dasatinib is not due to inhibition of Src kinase activity. The reduction in the osteoclast number could however also be mediated through the inhibiting effect of dasatinib on other tyrosine kinases, such as macrophage colony-stimulating factor receptor (c-fms) activation^66^. Signalling through c-fms is crucial for the proliferation and survival of osteoclasts. Likewise dasatinib also decrease expression of receptor activator of nuclear factor kappa B ligand (RANKL)^67^ - a crucial factor for osteoclast differentiation and activity. Additionally, the bone preserving effect observed can be mediated by an anabolic effect of dasatinib. Low dasatinib concentrations enhance osteoblastogenesis possibly through Src inhibition^42^. Among several Src family kinases only Src is synthetized and expressed in its activated form in osteoblasts. Src is therefore thought to have a dominant role in regulating osteoblast activity and knockdown of Src leads to decreased osteoblast proliferation and increased osteoblast differentiation^42^.

Only one other pre-clinical study has addressed the effect of multi-kinase inhibition on cancer-induced bone pain. As mentioned above, Felice and colleagues showed that daily oral administration of the multi-kinase inhibitor saracatinib, reversed thermal hyperalgesia, but not mechanical allodynia following inoculation of 3·10^3^ MRMT-1 cancer cells^34^. However, the study did not assess movement-evoked or weight-bearing induced pain and terminated the experiment 13 days post cancer cell inoculation, thereby only testing the analgesic potential in the early phase of pain development. The current study report the effect of Src inhibition in both the early stage and in in the more progressed stage of bone cancer pain and finds a delay in onset of pain-related behaviours post inoculation of 5·10^3^ MRMT-1 cells, thus supporting the finding by Felice *et al*.

The lack of anti-nociceptive effect of dasatinib in the advanced stage of the disease could be explained by the fast progressing disease phenotype. Bone micro-architectural results suggest that dasatinib slows down the pathological processes of cancer-induced bone pain by preserving the bone mass. However, the pain increases as the disease progresses towards the advanced stage. This might be due to a tumour-induced hyperinnervation or cancer-induced nerve compression/destruction, that counterbalance the bone preserving effect and consequently the indirect anti-nociceptive effect of the preserved bone mass is overruled by other pro-nociceptive mechanisms in the advanced stage of the disease. Similarly, other treatments with bone preserving effect such as bisphosphonates or denosumab do not prevent the progression of metastatic bone pain in advanced stages of the disease despite an antiresorptive effect^68, 69^. Also, studies have reported a non-persistent anti-nociceptive effect when administering drug candidates chronically in animal models of cancer-induced bone pain^70, 71^. Even morphine, which is commonly used in the clinic to treat cancer-induced bone pain have limited effect on pain-behaviour in the advanced stage of the disease. Animal studies have reported that the anti-nociceptive effect of morphine is reduced in cancer compared to other pain models^72^. Even though morphine is efficient in the early stages of cancer-induced bone pain, the anti-nociceptive effect is reduced in the advanced stage of the disease when administered in tolerable doses^73–75^.

A recent published randomized clinical trial also suggests a lack of effect of dasatinib on pain in late stage metastatic breast cancer. In the study by Schott el al. dasatinib was administered as 100 mg once daily, or 70 mg twice daily to patients with bone-predominant metastatic breast cancer. No effect on progression free survival or patient-reported pain was found^64^. This is comparable to our findings in the animal model and to the lack of analgesic effect observed when patients with advanced stages of bone metastases are treated with zoledronic acid and denosumab^68^.

In conclusion, in the current study we show that dasatinib delays onset of movement and non-evoked pain-related behaviour in the MRMT-1 Sprague Dawley rat model of cancer-induced bone pain. The effect is most likely due to disease modifying properties of dasatinib. Dasatinib, a multi-kinase inhibitor, preserves bone integrity in cancer-bearing animals leading to a delay in onset of nociceptive behaviour. These findings support a role of dasatinib in pathologies characterized by increased osteoclast activity, such as bone metastases. Indeed dasatinib has proven clinically efficient response in bone in combination treatment with zoledronic acid for the treatment of breast cancer metastases to bone^45^. However, dasatinib treatment does not eliminate development of pain-related behaviours in the advanced stage of the animal model, corresponding to the advanced-stage metastatic disease of the patients in the clinical trial by Schott *et al*., but merely delays the onset of pain. From a clinical point of view one might argue that the effect of dasatinib on bone preservation does not prove more beneficial compared to already clinically approved treatment options such as radiotherapy or bisphosphonates. Indeed zoledronic acid and dasatinib have similar mechanisms of action on rat bones^40^. Bisphosphonates inhibit osteoclast maturation and function and cause osteoclast apoptosis and compared to the anti-neoplastic dasatinib, they generally cause less severe side effects. Furthermore, several bisphosphonates, e.g. zoledronic acid and ibandronate have been proven effective in reducing bone pain in patients with bone metastases and maintained low bone pain scores for up to several years^76^.

## Methods

### Animals and housing

6 weeks old female Sprague-Dawley rats weighing 150–170 g (Taconic M&B A/S, Denmark) were group-housed in cages of 5 under a 12:12 h light-dark-cycle, in a climate-controlled room, with ad libitum access to food and water. The animals were allowed to acclimatize 1–2 weeks before starting the experiments. The general health condition of each rat was checked regularly and the body weight was measured every third day all through the experiment. Experiments were approved by the Danish Animal Experiments Inspectorate, The Danish Veterinary and Food Administration, Ministry of Environment and Food (license no. 2014-15-0201-00031 C4) and conducted under the guidelines of the International Study of Pain^77^. All experiments and data analysis were blinded for the researchers.

### Cell line culturing and preparation for surgery

The mammary rat metastasis tumour cells-1 transfected with the luciferase gene, MRMT1-*luc2*, were cultured as previously described for the MRMT-1 cells^78, 79^. Briefly, MRMT1-*luc2* were cultured in RPMI-1640 free of glutamine and phenol red (Gibco, Life Technologies Europe BD, Denmark) with 5% penicillin-streptomycin-glutamine (10,000 Units/mL, 10,000 µg/mL and 29.2 mg/mL respectively) (Gibco, Life Technologies Europe BD, Denmark) and 10% heat-inactivated foetal bovine serum (Gibco, Life Technologies Europe BD, Denmark) and maintained at 37 °C with 5% CO_2_. On the day of cancer inoculation the cells were washed twice with phosphate buffered saline (no Mg^2+^, no Ca^2+^), trypsinized with 0.1% w/v Trypsin-EDTA (Gibco, Life Technologies Europe BD, Denmark), centrifuged at 220 g for 3 minutes, washed twice with Hank’s balanced salt solution (HBSS) (Gibco, Life Technologies Europe BD, Denmark), counted and suspended in a HBSS solution of 5·10^5^ cells/ml. The cells were kept on wet ice until use. The cells prepared for *in vitro* cell assays were washed with medium instead of HBSS.

### Cancer-induced bone pain model

The cancer-induced bone pain model was performed as previously described^78^. Shortly, the rat was anaesthetized with 2–4% inhalation isoflurane (Baxter A/S, Allerød, Denmark), administered Rimadyl (s.c. 5 mg/kg, Pfizer, Denmark) and placed on its back and confirmed free of reflexes. The anterior surface of the right tibia was shaved and disinfected with 70% v/v ethanol. A small incision was made with a scalpel on the anterior medial side of the tibia and the tibia was thereafter cautiously exposed and cleaned with a cotton swab. Using a 0.7 mm dental drill a hole was made in the tibia and a thin polyethylene tube (outer diameter 0.61 mm) (Smiths medical, Denmark) inserted 1 cm into the medullary cavity towards the proximal part of the bone and 5·10^3^ cells in 10 µL HBSS was injected using a Hamilton syringe. Sham animals were injected with 10 µL HBSS alone. After 1 minute the polyethylene tube was removed and the hole closed with dental cement (IRM, Dentsply, Konstanz, Germany), the wound cleaned with isotonic saline, closed with metal clips (Kruuse, Denmark) and applied topically with 2% lidocaine gel (AstraZeneca, Denmark). The animal was placed to awake under a heat lamp and thereafter placed in their home cage.

### *In vivo* experiments

#### Treatment study

52 female Sprague-Dawley rats were randomized according to bodyweight into the following five treatment groups. Group 1, 2 and 3 was inoculated with MRMT1-*luc2* and received vehicle (n = 10), 5 mg/kg dasatinib (n = 11) or 15 mg/kg dasatinib (n = 11), respectively. Group 4 and 5 underwent sham surgery and were treated with vehicle (n = 10) or 15 mg/kg dasatinib (n = 10), respectively. The vehicle was a sterile mix of 80 mM citric acid and 80 mM sodium citrate with a final pH of 3.1. Drug or vehicle treatment was administered every morning, minimum 60 minutes before behavioural testing, by oral gavage starting from day 7 post inoculation.

The rats were introduced to the behavioural tests twice before baseline measurements in order to avoid stress-induced bias of the behaviour readouts. Behavioural tests were performed two days before surgery and on day 7, 10, 13, 16, 18, 20, 22 and 23 post inoculation of MRMT1-*luc2* mammary carcinoma cells.

#### Timeline study

75 female Sprague-Dawley rats were assigned into the following three groups: naïve (n = 15), cancer-operated (n = 30) or sham-operated (n = 30). One third of the animals from each group were euthanized on post inoculation day 7, 12 and 16. Behavioural tests and *in vivo* imaging were performed on day 7, 12 and 16.

#### Limb use test

All animals from one home cage was transferred to a transparent empty plastic box (500 mm × 300 mm × 500 mm) and allowed to familiarize themselves to the new environment for 10 minutes. After the habituation all rats were observed individually in the plastic box for 3 minutes and the gait of the affected leg was scored using an in-house scoring system validated for inter-experimenter variability. The scale ranged from 3 to 0 where; 3 was normal use of the cancer-bearing limb; 2 was a slight limp but normal body distribution; 1 was a significant limp and a shift in body distribution toward the healthy limb; 0 was a partly lack of use of the cancer-bearing limb defined as holding the hind limb aloft while in locomotor activity and/or sitting.

#### Weight-bearing test

The rats were placed in an incapacitance tester (MJS Technology Ltd., Buntingford, Herfordshire, UK) with the hind legs resting on two separate weights while the front legs were resting on the wall of the incapacitance tester. The individual load of each hind limb was measured for 4 seconds and measurements performed in triplicates. The average weight-bearing ratio was calculated as the amount of weight placed on the cancer-bearing leg divided by the total amount of weight put on both legs.

### Bioluminescent imaging

The rat was anesthetized with 2–3% inhalation isoflurane (Baxter A/S, Allerød, Denmark), and injected intra-peritoneal with 40 mg/kg D-luciferin (PerkinElmer, Denmark) dissolved in PBS. 10 minutes after injection the animal was transferred to the IVIS® Lumina XR (Caliper Life Sciences, USA) and placed on its back. Bioluminescent image settings were as followed; binning: M(4); F/stop: 1 and exposure times was adjusted according to the signal and varied from 1 s to 120 s. Three images were taken per animal and the animal was repositioned between each image in order to avoid bias. The ROI threshold was set to 35% and the signal was recorded in radiance as photons/s/cm^2^/sr.

### X-ray capturing and analysis

An x-ray image of the ipsilateral tibia was taken immediately following the bioluminescent images. The tibia was placed next to an aluminium wedge which was used as a control for comparison of the relative bone density of the tibia between images. The x-ray image was analysed using NIH ImageJ (ImageJ, 1.47 v). The greyscale value of the proximal trabecular part of the tibia was measured and an average of the surrounding tissue was subtracted and normalized to the constant aluminium wedge.

### Microcomputed tomography

Sprague-Dawley rats treated with dasatinib or vehicle were briefly anesthetized with 4% inhalation isoflurane and euthanized by decapitation. The ipsilateral tibia was removed and fixated in 4% paraformaldehyde (PFA) for 7 days and thereafter stored in phosphate buffered saline containing 0.1% PFA and 0.1% NaN_3_ at 4 °C. In order to quantify the tumour-induced osteolysis, the proximal tibia section was scanned with a high-resolution microcomputed tomographic (μCT) system (vivaCT40; Scanco Medical AG, Brüttisellen, Switzerland), with a spatial 3-dimensional (3D) reconstruction of cubic voxel sizes of 12.5 × 12.5 × 12.5μm^3^. Each 3D image dataset consisted of approximately 210 μCT slide images of which 100 slide images (1250μm) were used for analysis of bone tissue (2048 × 2048 pixels) with 16-bit grey levels. Bone volume fraction (BV/TV), bone surface to bone volume ratio (BS/BV), and trabecular separation (TbSp) were calculated based on assumption-free 3D methods^80^.

### Western blot

#### Timeline study

At the end of the experiments all rats were anesthetized with 4% isoflurane and euthanized by decapitation. The spine was exposed and cut free at the sacral and thoracic segments and the spinal cord was flushed out from the caudal side using an 18 G blunt needle and sterile isotonic saline. The segment of the spinal cord correlating to L2-L5 was isolated, divided into an ipsi- and contralateral part, snap frozen on dry ice, and stored at −80 °C.

#### Treatment study

After decapitation, the back of the rat was disinfected with 70% v/v ethanol and a mid-sagittal incision was made with a scalpel, the skin removed and the spine made visible. The tissue surrounding the spinal cord was removed (Allgaier Instruments GmbH, Frittlingen, Germany) thereby exposing the spine. Using a scissor a transversal cut was made at the rostral end of the spine in order to expose the spinal cord. The dorsal part of the spine was then removed from the thoracic part and continuing to the end of the lumbar section. The segment of the spinal cord correlating to L2-L5 was isolated, divided into ipsi- and contralateral part, snap frozen on dry ice, and afterwards stored at −80 °C.

Each spinal cord segment was weighed and homogenized in a 1 g to 30 mL ratio of solubilisation buffer pH 6.8 (50 mL of pre-solubilisation buffer containing 0.985% Tris-Hcl, 2% (w/v) Sodium Dodecyl Sulphate and demineralized H_2_O mixed with 1 tablet cOmplete™ Protease Inhibitor Cocktail (Roche Diagnostics GmbH, Mannheim) and 5 tablets PhosSTOP™ Phosphatase Inhibitor Cocktail Tablets (Roche Diagnostics GmbH, Mannheim)). After the first homogenization round the samples were stored at 4 °C for 3 h, homogenized again, stored for 30 min at 4 °C and finally centrifuged for 30 min at 20,000 g at 4 °C. The supernatant was collected and stored at −80 °C until use.

Samples were thawed on wet ice, diluted 1:1 in 2x loading buffer with 5% mercaptoethanol and boiled for 5 min. 12 µL of each sample were loaded per well on a 7.5% Criterion™ TGX Stain-Free™ Protein Gel, 26 well, 15 µl (Biorad, Hercules, CA) and blotted onto a PVDF membrane (Biorad, Hercules, CA) using the Biorad Trans-Blot® Turbo™ Transfer system set to 2.5 A, 25 V for 35 min. The membrane was washed with TBS-T and blocked with 5% (w/v) Bovine Serum Albumin (Sigma-Aldrich, Brøndby, Denmark) which was also used for incubation with all antibodies. Blots were incubated overnight at 4 °C and incubated with the following antibodies: Phospho-Src Family (Tyr416) Antibody #2101, 1:100 (Cell Signaling Technology, Massachusetts, USA); c-Src Antibody (SRC 2): sc-18 1:1000 (Santa Cruz Biotechnology, Texas, USA); Phospho-NMDAR2B (Tyr1472) Antibody #4208 1:1000 (Cell Signaling Technology, Massachusetts, USA); NMDAR2B Antibody #4207 1:1000 (Cell Signaling Technology, Massachusetts, USA). After washing with TBS-T blots were incubated 1 h with (1:2,000) Goat Anti-Rabbit IgG H&L (HRP) ab6721 (Abcam) and developed for 1 minute in the Western Lightning ECL Pro kit (PerkinElmer). The signal was detected and subsequently analysed in the Molecular Imager® ChemiDoc™ XRS Imaging System with Quantity One software (Bio-Rad).

### ELISA analyses of TRACP 5b in rat serum

Serum samples were collected from the rats of the treatment study on day 6, 12 and on the day of euthanasia. Animals were fasted for 6 hours before serum collection. 600 µL blood was collected from the sublingual vein, stored in microcentrifuge tubes at room temperature for 15–30 min, and thereafter centrifuged at 4600 rpm for 10 min at 4 °C. The supernatant was collected and stored at −80 °C until analysis.

Osteoclast-derived tartrate resistant acid phosphatase form 5b (TRACP 5b) levels were quantified in rat serum by ELISA using the RatTRAP^TM^ Assay, SB-TR102 (Immunodiagnostics Systems Nordic a/s, Herlev, Denmark). The assay was performed as described by the manufacturer (Immunodiagnostics Systems Nordic a/s, Herlev, Denmark). Samples were diluted 1:4 in isotonic saline, run in duplicates and the absorbance measured at 405 nm using a Versa Max microplate reader (Molecular Devices, LLC., California, USA).

### Cell viability assays

Cell proliferation and viability was investigated *in vitro* using the CellTiter 96® Non-Radioactive Cell Proliferation Assay (MTT) (Promega, Nacka, Sweden), cell proliferation ELISA, BrdU (colorimetric) kit (Roche Diagnostics GmbH, Mannheim, D), and the RealTime-Glo™ MT Cell Viability Assay (Promega, Nacka, Sweden). For all assays MRMT1-*luc2* cells were harvested, as described above, and 1000 cells/well was plated in a 96 well plate in 99 µL RPMI-1640 no glutamine, no phenol red medium (Gibco, Life Technologies Europe BD, Denmark). After incubation at 37 °C with 5% CO_2_ for 24 h 1 µL dasatinib was added in decreasing concentrations (3·10^−4^–205 µM) dissolved in dimethylsulfoxide (DMSO) and incubated for additional 48 h. The MTT assay was performed three times and all measurements were performed in quadruplets. Incubation time with dye in the MTT assay was set to 3 h. All measurements for RealTime-Glo™ and the BrdU assays were performed in triplicates. The assays were performed as described by the manufacturers. For the BrdU assay the labelling time for BrdU was set to 8 h, FixDenat incubation was 30 min and Substrate solution incubation time was 5 min. In the RealTime-Glo™ assay the Continuous-Read Method was chosen, and the bioluminescent measured at 1000 ms in white, flat bottomed 96 well plates. For all assays the EC_50_ values were calculated using GraphPad Prism® software, version 6 (GraphPad Software, La Jolla, CA, USA)

### Statistical analyses

Statistical analyses were performed in GraphPad Prism (version 6; GraphPad Software, CA, USA). A 95% confidence interval was chosen as a measure of statistical significance, p > 0.05. The multiplicity adjusted p value is noted in the text. In the figures, the asterisks denote the following level of significance: *p < 0.05; **p < 0.01; ***p < 0.001; ****p < 0.0001. Data are displayed as means and the standard error of the mean ( ± SEM). Behavioural data were analysed by a two-way repeated-measures ANOVA followed by Bonferroni’s multiple comparison test for significance at individual time points. Changes in serum TRACP 5b marker and bone microarchitecture were analysed by a one-way ANOVA followed by Bonferroni’s post-hoc test. For western blot data outliers were identified by the ROUT method which identify outliers from nonlinear regression, Q was set to 1%. Western blot results where then analysed by one-way ANOVA followed by Bonferroni’s post-hoc test. If an animal, due to reaching of a humane endpoint (defined as limb use score of 0 or weight loss above 20%), was euthanized before day 23, the last measurements were carried forward to the following measuring days and used for data analyses.

## Electronic supplementary material

Supplementary Information File
