# Author Correction: The Src family kinase inhibitor dasatinib delays pain-related behaviour and conserves bone in a rat model of cancer-induced bone pain

**DOI:** 10.1038/s41598-019-43721-6

**Published:** 2019-05-09

**Authors:** Camilla Kristine Appel, Simone Gallego-Pedersen, Line Andersen, Sophie Blancheflor Kristensen, Ming Ding, Sarah Falk, Manasi Sayilekshmy, Charlotte Gabel-Jensen, Anne-Marie Heegaard

**Affiliations:** 1 University of Copenhagen, Faculty of Health and Medical Sciences, Department of Drug Design and Pharmacology, Copenhagen, 2100 Denmark; 2 Odense University Hospital, University of Southern Denmark, Institute of Clinical Research, Department of Orthopaedics and Traumatology, Odense, 5320 Denmark; 3 University of Copenhagen, Faculty of Health and Medical Sciences, Department of Pharmacy, Copenhagen, 2100 Denmark

Correction to: *Scientific Reports* 10.1038/s41598-017-05029-1, published online 06 July 2017

The Acknowledgements section in this Article is incomplete.

“The authors thank Camilla Skånstrøm Dall and Tina Maria Estrup Axen for technical assistance. This project was supported by *Advokat Bent Thorbergs Fond*.”

should read:

“The authors thank Camilla Skånstrøm Dall and Tina Maria Estrup Axen for technical assistance. This project was supported by *Advokat Bent Thorbergs Fond*. This project has received funding from the European Union’s Horizon 2020 research and innovation programme under the Marie Skłodowska-Curie grant agreement No 642720.”

