# Supplementary material for: The Src family kinase inhibitor dasatinib delays pain-related behaviour and conserves bone in a rat model of cancer-induced bone pain
Source: Sci Rep. 2017 Jul 6;7:4792. doi: 10.1038/s41598-017-05029-1 (PMC5500481; doi:10.1038/s41598-017-05029-1)

The Src family kinase inhibitor dasatinib delays pain-related behaviour and conserves bone in a rat model of cancer-induced bone pain

Camilla Kristine Appel[1], Simone Gallego-Pedersen[1], Line Andersen[1], Sophie Blancheflor Kristensen[1], Ming Ding[2], Sarah Falk,[1], Manasi Sayilekshmy[1], Charlotte Gabel-Jensen[3], Anne-Marie Heegaard[1]*.

[1]University of Copenhagen, Faculty of Health and Medical Sciences, Department of Drug Design and Pharmacology, Copenhagen, 2100, Denmark.

[2]Odense University Hospital, University of Southern Denmark, Institute of Clinical Research, Department of Orthopaedics and Traumatology, Odense, 5320, Denmark

[3]University of Copenhagen, Faculty of Health and Medical Sciences, Department of Pharmacy, Copenhagen, 2100, Denmark

*amhe@sund.ku.dk

Supplementary information

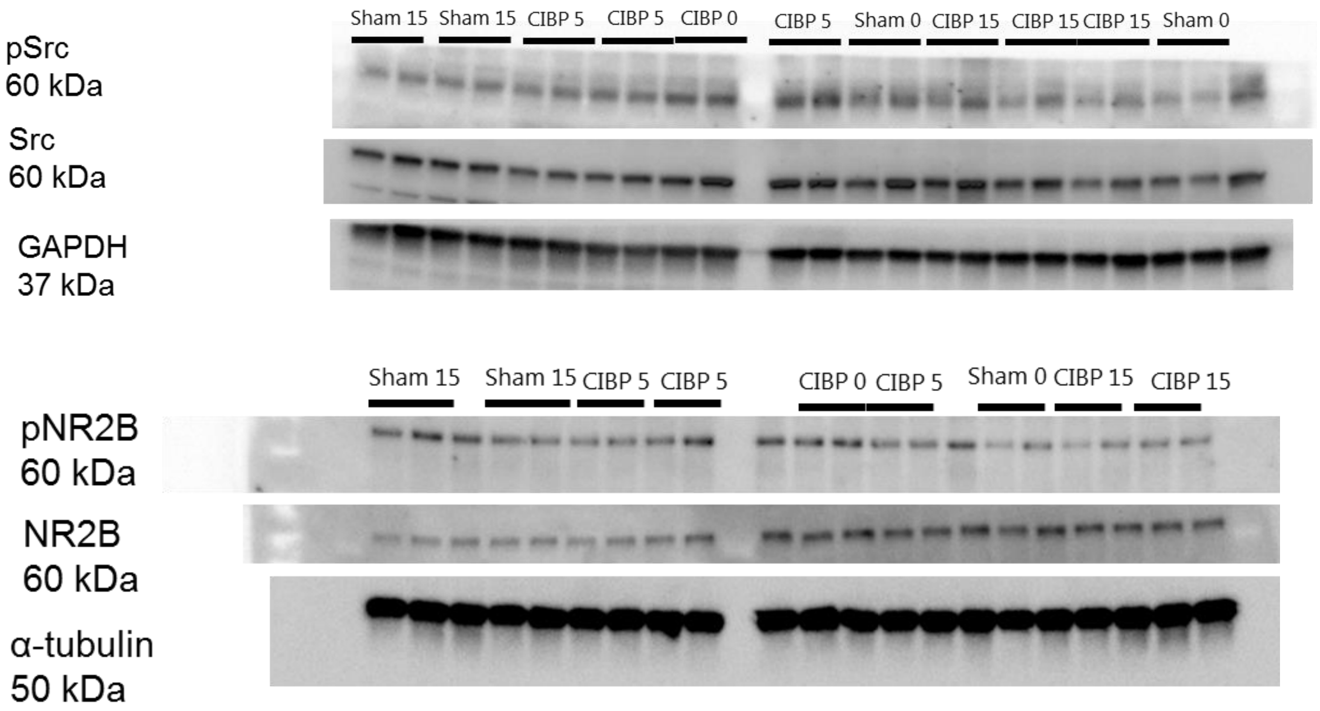

Supplement: Supplementary file 1 — Supplementary Information File [file 41598_2017_5029_MOESM1_ESM.pdf]
